# Polyploidization for the Genetic Improvement of *Cannabis sativa*

**DOI:** 10.3389/fpls.2019.00476

**Published:** 2019-04-30

**Authors:** Jessica L. Parsons, Sara L. Martin, Tracey James, Gregory Golenia, Ekaterina A. Boudko, Shelley R. Hepworth

**Affiliations:** ^1^Canopy Growth Corporation, Smiths Falls, ON, Canada; ^2^Department of Biology, Carleton University, Ottawa, ON, Canada; ^3^Ottawa Research and Development Centre, Agriculture and Agri-Food Canada, Ottawa, ON, Canada

**Keywords:** *Cannabis sativa*, tissue culture, polyploidy, tetraploid, flow cytometry, THC, CBD, terpenes

## Abstract

*Cannabis sativa L.* is a diploid species, cultivated throughout the ages as a source of fiber, food, and secondary metabolites with therapeutic and recreational properties. Polyploidization is considered as a valuable tool in the genetic improvement of crop plants. Although this method has been used in hemp-type Cannabis, it has never been applied to drug-type strains. Here, we describe the development of tetraploid drug-type Cannabis lines and test whether this transformation alters yield or the profile of important secondary metabolites: Δ^9^-tetrahydrocannabinol (THC), cannabidiol (CBD), or terpenes. The mitotic spindle inhibitor oryzalin was used to induce polyploids in a THC/CBD balanced drug-type strain of *Cannabis sativa*. Cultured axillary bud explants were exposed to a range of oryzalin concentrations for 24 h. Flow cytometry was used to assess the ploidy of regenerated shoots. Treatment with 20–40 μM oryzalin produced the highest number of tetraploids. Tetraploid clones were assessed for changes in morphology and chemical profile compared to diploid control plants. Tetraploid fan leaves were larger, with stomata about 30% larger and about half as dense compared to diploids. Trichome density was increased by about 40% on tetraploid sugar leaves, coupled with significant changes in the terpene profile and a 9% increase in CBD that was significant in buds. No significant increase in yield of dried bud or THC content was observed. This research lays important groundwork for the breeding and development of new Cannabis strains with diverse chemical profiles, of benefit to medical and recreational users.

## Introduction

Cannabis (*Cannabis sativa* L.) has been used as a source for fiber, food, medicine, and recreation for over 5000 years (Thomas and Elsohly, 2016). Recently, there has been renewed interest in Cannabis due to its many medicinal effects, particularly the treatment of epilepsy, pain, and nausea associated with cancer treatment (Andre et al., 2016; Thomas and Elsohly, 2016). The government of Canada recognizes over two dozen conditions for which Cannabis is an effective treatment (Health Canada, 2018). While there are hundreds of different active metabolites present in Cannabis, two cannabinoids are present in high concentrations, and are generally considered to be the most important: Δ^9^-tetrahydrocannabinol (THC) and cannabidiol (CBD). THC is responsible for the well-known psychoactive properties of Cannabis whereas non-intoxicating CBD is widely used for pain, anxiety, depression, and sleep disorders (Andre et al., 2016; Corroon and Phillips, 2018). Another group of important chemicals is the terpenes, which contribute to the smell and taste of Cannabis products, but also function as active metabolites with therapeutic properties (Russo, 2011; Andre et al., 2016). All of these metabolites are produced and stored within glandular trichomes that mainly develop on the inflorescence of the plant (Marks et al., 2009; Andre et al., 2016).

Several medicinal cannabinoid preparations are available including Marinol^®^, a synthetic THC preparation for treatment of anorexia in AIDS patients, Sativex^®^, a mouth spray with THC and CBD for treatment of multiple sclerosis pain, and Epidiolex^®^ for treatment of pediatric seizure disorders (Corroon and Phillips, 2018; Health Canada, 2018). However, using whole Cannabis can be more effective than the single ingredient preparations for some conditions due to the synergy between multiple phytochemicals. In particular, CBD and the terpenes can modulate the effects of THC (Wilkinson et al., 2003; Brenneisen, 2007; Russo, 2011; Andre et al., 2016). For example, CBD can inhibit the metabolism of THC to the more potent 11-OH-THC upon ingestion (Brenneisen, 2007), and can reduce some of the negative side-effects of THC like anxiety, hunger, and sedation (Mechoulam et al., 2002; Russo, 2011; Andre et al., 2016). Therefore, developing a wider variety of Cannabis strains may be preferable to new formulations of the active ingredients.

Historically, new Cannabis strains have been developed through conventional breeding methods. However, these methods can be imprecise, and require several generations before the desired traits are obtained and a stable strain is produced. One strategy to accelerate breeding development is a chromosome doubling event called polyploidization (Sattler et al., 2016). We therefore investigated this method for developing improved Cannabis strains.

Polyploidization is common in the plant kingdom and has been associated with increased genetic diversity in some plant lineages (Comai, 2005). Desirable consequences of polyploidy for plant breeding include the buffering of deleterious mutations, increased heterozygosity, and hybrid vigor (Sattler et al., 2016). Consequently, polyploids often have phenotypic traits that are distinct from diploids, including larger flowers or leaves (Dermen, 1940; Rêgo et al., 2011; Trojak-Goluch and Skomra, 2013; Sattler et al., 2016; Talebi et al., 2017). Increases in active metabolite concentration in tetraploids are reported for numerous medicinal plants including *Artemisia annua* (Wallaart et al., 1999), *Papaver somniferum* (Mishra et al., 2010), *Datura stramonium* (Berkov and Philipov, 2002), *Thymus persicus* (Tavan et al., 2015), *Echinacea purpurea* (Abdoli et al., 2013), and *Tanacetum parthenium* (Majdi et al., 2010). The introduction of some of these polyploid traits would be beneficial for the cultivation of Cannabis. Cannabis is diploid plant with 20 chromosomes (Van Bakel et al., 2011). Doubling the chromosome set should allow more flexibility to increase potency or tailor the cannabinoid ratios. A handful of studies support the theory that polyploid Cannabis might have higher potency, although the results are mixed, with some studies finding decreases in THC (Clarke, 1981; Bagheri and Mansouri, 2015; Mansouri and Bagheri, 2017). However, these studies were conducted with hemp. The effects of polyploidization on drug-type Cannabis strains is unknown.

Polyploidy can be induced through application of antimitotic agents to seeds, seedlings, *in vivo* shoot tips, or *in vitro* explants (Dermen, 1940; Petersen et al., 2003; Talebi et al., 2017). However, drug-type Cannabis strains are not genetically stable when propagated through seeds, and while there has been little success in regenerating Cannabis shoots from callus, the propagation of high THC drug-type Cannabis in tissue culture using nodal explants has been described. These plants have been shown to be genetically and chemically stable through 30 rounds of tissue culture propagation (Lata et al., 2009, 2016).

Here, we describe an effective method for generating Cannabis tetraploids from axillary bud explants and the subsequent analysis of polyploidy effects on growth, yield, and phytochemistry in a drug-type strain. This research lays important groundwork for the development of improved Cannabis strains and novel germplasm for breeding efforts.

## Materials and Methods

### Plant Material

*Cannabis sativa* L. (Cannabis) plants were provided by Canopy Growth Corporation. All plants were cultivated in an indoor facility in growth rooms controlled for light, temperature, and humidity (Tweed Inc., Smiths Falls, ON, Canada). Mother plants for sampling were grown under 18 h of light. Plants were watered daily with a nutrient solution (General Hydroponics Cocotek Grow A/B). Two commercial non-inbred strains were tested: one THC dominant indica strain (Strain 1), and one balanced THC/CBD indica-dominant hybrid strain (Strain 2).

### Culture Methods

Nodal segments containing young axillary buds with no fully expanded leaves were harvested from a healthy mother plant. Explants were taken from a single mother plant of each genotype to ensure consistency. Fan leaves and stipules were removed from the axillary bud, and the stem was cut at a 45° angle leaving approximately 5 mm of stem below the axillary bud. Explants were sterilized in a solution of 2% sodium hypochlorite (diluted household bleach) and 0.1% (v/v) Tween-20 for 5 min and then rinsed in sterile distilled water three times for 1 min prior to inoculation on culture medium.

Sterilized axillary bud explants were cultured in round-bottom glass culture vessels (25 × 150 mm test tubes with plastic caps, PhytoTechnology Laboratories C2093 and C1805) containing 20 mL of shooting media. The shooting media was composed of 1× Murashige and Skoog (MS) basal medium with vitamins (PhytoTechnology Laboratories, M519) supplemented with 30 g L^-1^ sucrose (VWR SS1020) and 0.3 g L^-1^ charcoal (PhytoTechnology Laboratories, C325) adjusted to pH 5.75 and solidified with 8.0 g L^-1^ agar (PhytoTechnology Laboratories, A296). Plant growth regulators were added after autoclaving, 0.1 mg L^-1^ α-naphthaleneacetic acid (PhytoTechnology Laboratories, N600) and 0.4 mg L^-1^ kinetin (PhytoTechnology Laboratories, K750). Sterile shoots emerged after 1–5 months. Plantlets were subcultured onto fresh media every month or as required. Plantlets with elongated shoots (taller than 2.5 cm) were moved to larger glass vessels with vented caps (62 × 95 mm glass jar, PhytoTechnology Laboratories C2099 and C176) containing 50 mL of rooting media. Rooting media was the same composition as shooting media (1× MS, sucrose, charcoal) except contained 1.0 mg L^-1^ indole-3-butyric acid (PhytoTechnology Laboratories, I538) and was solidified with 4.0 g L^-1^ gelzan (PhytoTechnology Laboratories, G3251). Roots typically emerged after 3–5 weeks. If plantlets rooted in the shooting media they were not moved. All cultures were incubated at 24°C under white fluorescent lighting (16 h photoperiod, average light intensity 75 μmol m^-2^s^-1^).

Plantlets with an established root system (about 3 weeks after root emergence) were carefully removed from the medium, rinsed under lukewarm tap water to remove debris, and transplanted into soil to acclimatize. Plants were placed in 500 mL plastic pots containing high porosity growing medium with mycorrhizae (Pro-Mix, Product 20381) and transferred to a temperature and humidity-controlled growth room (24°C and 40% relative humidity). Plants were grown under white fluorescent lighting (18 h photoperiod; average light intensity 115 μmol m^-2^s^-1^). The pots were covered with a humidity dome for the first week or two, venting the domes near the end to gradually bring down the humidity. After the removal of humidity domes, plants were watered daily with a fertilizer solution (General Hydroponics Cocotek Grow A/B, prepared to an electrical conductivity of 1.0 mS cm^-1^).

### Oryzalin Treatments to Induce Polyploids

Disinfected axillary buds (10 replicates per genotype) were placed into treatment media containing 0 (control), 50, 100, or 150 μM oryzalin (3,5-dinitro-N^4^,N^4^-dipropylsulfanilamide) to induce polyploidy (PhytoTechnology Laboratories, O630). A second trial was conducted using 0 (control) and 20, 40, or 60 μM oryzalin concentrations (8 replicates per genotype). The treatment media was prepared by diluting a stock solution (37.5 mM oryzalin in 80% ethanol) into 25 mL of liquid MS media containing 30 g L^-1^ sucrose (pH 5.75). The cultures were covered in tin foil to prevent light degradation of the oryzalin, then rocked on an orbital shaker (150 rpm). After 24 h, the oryzalin solution was removed, and axillary buds were rinsed three times with sterile distilled water containing 1 mL L^-1^ of the broad-spectrum biocide Plant Preservative Mixture (Plant Cell Technology). The axillary buds were placed on shooting media and cultured as described above. Once explants had recovered and grown at least three leaves, one leaf per plant was sampled for flow cytometric ploidy analysis. If an explant had developed more than one primary stem, one leaf per branch was tested. Plants determined to be tetraploid were transplanted into soil and grown to maturity.

### Flow Cytometric Analysis

Total nuclear DNA content was assessed by flow cytometry. Young leaves were collected from healthy Cannabis mothers or culture plants and stored in damp paper towel on ice for up to 24 h prior to analysis. All materials and samples were kept on ice throughout preparation. Leaf samples of 0.5 cm^2^ were chopped with a razor blade in a Petri dish containing 750 μL of ice-cold lysis buffer LB01 (Doležel et al., 1989). The suspension was passed through a 30 μm nylon mesh filter to isolate the nuclei (Celltrics). The filtrate was treated with 50 μL of RNAase (1 mg mL^-1^) and stained with 250 μL of propidium iodide (0.1 mg mL^-1^) for 30 min in the dark. Ploidy was analyzed on a Gallios flow cytometer (Beckman Coulter, ON, Canada). The stained nuclei were analyzed with method parameters 465 V and for a maximum of 120 s capturing data for at least 1000 nuclei per sample.

Cannabis leaf samples were co-chopped with radish *Raphanus sativa* “Saxa” (2*n* = 2× = 16 chromosomes, 2C = 1.11 pg) as an internal standard (Doležel et al., 1992; Martin et al., 2015). Relative DNA content was determined using fluorescence peak area (585/42 nm detector) and fluorescence peak means, coefficients of variation, and nuclei numbers were measured using the flow Ploidy package in R (Martin et al., 2015; Smith et al., 2018). Genome sizes were measured on three non-consecutive days to ensure accuracy (Martin et al., 2015).

### Cytological Techniques

The ploidy level of the diploid mother plant and *in vitro* polyploid plants was confirmed by chromosome count. Young healthy roots were harvested from plants and rinsed with tap water to remove all traces of media. The roots were placed in a 1.5 ml microcentrifuge tube with water and pretreated with nitrous oxide for 1 h in a custom-built pressurized chamber at 160 psi to accumulate metaphase cells (Andres and Kuraparthy, 2013). The roots were then fixed in a 3:1 ethanol:acetic acid mixture at room temperature for 24–48 h. The root tips were digested in 1 M HCl for 5 min at 60°C and then rinsed with ice-cold water three times. The root tip cells were then excised and macerated on a microscope slide following the squash method of Tsuchiya and Nakamura (1979) and stained with a drop of 2% acetocarmine. Cells were imaged using a compound microscope (Zeiss Lab A1) with color camera (Zeiss Axiocam 105). Chromosomes were counted in at least three root tip cells per genotype.

### Phenotypic Analyses

Growth parameters were measured for diploid and tetraploid clones to assess the effects of polyploidy. To generate material for this analysis, healthy plants in tissue culture were transferred to soil and grown into mother plants.

Fifteen cuttings from each mother were rooted in peat-based foam plugs (Grow-Tech LLC., 72R plugs) using Stim Root #1 rooting powder (Plant Prod, ON, Canada). The clones were covered with a humidity dome and irrigated with a nutrient solution (General Hydroponics Cocotek Grow A/B, prepared to an electrical conductivity of 1.0 mS cm^-1^) until roots were established. Most clones were successfully rooted after 3 weeks at which point the humidity domes were removed. Plants were grown under white fluorescent lighting (18 h photoperiod; average light intensity 115 μmol m^-2^s^-1^). Half-lighting was applied during the early stages of clone rooting.

After 5 weeks, nine or ten healthy clones per genotype were transplanted into one-gallon pots containing high porosity growing medium with mycorrhizae (Pro-Mix, Product 20381). Particularly tall clones had their lower stems trimmed and were buried deeper than the shorter ones, a common practice in Cannabis cultivation to ensure uniform light intensity and water use. Plants were watered daily with a nutrient solution: General Hydroponics Cocotek Grow A/B during the vegetative phase and General Hydroponics Cocotek Bloom A/B during the flowering phase (both prepared to an electricial conductivity of 2.5 mS cm^-1^). Plants were grown for 4 weeks in the vegetative growth phase (18 h photoperiod, average light intensity 220 μmol m^-2^s^-1^ under metal halide lamps) and for 9 weeks in the flowering phase (12 h photoperiod, average light intensity 485 μmol m^-2^s^-1^ under high pressure sodium lamps). After 2 weeks of vegetative growth, the apical portion of the plant was removed to leave six remaining lateral branches (topping). Subsequently, the ploidy level of tetraploid clones was retested by flow cytometry. In the final week of vegetative growth, the plants were transplanted into two-gallon pots and moved to the flowering room to acclimatize to the higher light intensity before exposure to the flowering light cycle. Following this switch, the plants were pruned as required to remove excess leaves and small stems to ensure adequate light penetration and air flow in the canopy to discourage pathogens (weeks 1, 3, and 4 of flowering).

Growth parameters were measured once a week starting at the time of clone transplant to one-gallon pots. Specifically, plant height (from soil to the highest apical meristem), stem diameter (1 inch above soil level), cumulative length of all primary lateral branches (measured from node to apical meristem), and width of central leaflets (at widest point including teeth using three mature fan leaves per plant) were measured. During the flowering phase, measurements were taken every 2 or 3 weeks on account of slower growth. Plants were harvested after 9 weeks of flowering corresponding to 13 weeks of growth following clone transplant to one-gallon pots.

Upon harvesting, the plants were weighed whole and then separated into bud, leaf, and stem portions. Each portion was weighed individually. The bud samples were composed of equal portions of cola and popcorn buds (buds from the top and bottom of a stem, respectively). The leaf samples were composed of equal portions of fan leaves (large vegetative leaves) and sugar leaves (small reduced leaves that grow on the inflorescence). The samples were set on trays to dry in a climate-controlled room for 1 week. The weight of the dried bud material was measured to determine the final yield.

### Stomata Characteristics

Nail polish impressions were used to compare the size and density of stomata on the abaxial surface of diploid and tetraploid mature fan leaves (Grant and Vatnick, 2004). The impressions were dried overnight and then viewed under a compound microscope with color camera as described above. The number of stomata per field of view under the 40x objective was used to calculate the density of stomata in eight different images. In each image, the length and width of three stomata guard cells were measured using Zeiss ZEN blue imaging and analysis software. The size of the image was measured to calculate the number of stomata per mm^2^.

### Trichome Density Measurements

Two weeks before plants were harvested, trichome density was measured on diploid and tetraploid sugar leaves (the reduced leaves that grow in the inflorescence). Three large stems per plant were selected at random and the 4^th^ leaf from the apex was harvested. The adaxial surface of the central leaflet was imaged at its widest point under 10× magnification using a camera lens attachment on a stereoscope (Zeiss Stemi DV4). A ruler in each photo was used as a scale. The stalked glandular trichomes were counted within a 16 mm^2^ area of each leaf on one side of the midrib. For very small leaves, a 9 mm^2^ area was used to calculate the trichome density.

### Chemotype Analysis

Bud and leaf portions of diploid and tetraploid plants were sampled for analysis of cannabinoid and terpene content. For cannabinoid analysis, 0.5 g of dried, homogenized tissue was placed in a glass test tube with 10 mL of extraction solution (1:9 solution HPLC grade chloroform and methanol). The samples were then sonicated for 30 min and spun down. The extraction solution was filtered and diluted 10× in HPLC grade methanol. Cannabinoid samples were prepared in duplicate. For terpene analysis, 10 mg of homogenized sample was placed directly into a headspace vial.

Twelve cannabinoids were assessed using an Agilent 1200 HPLC with a diode array detector. Twenty-three terpenes were assessed using an Agilent 7820A/7890B gas chromatograph system with a flame ionization detector. Chemstation software [Open LAB CDS Chemstation Edition Rev. A.02.02(1.3)] was used to analyze the data. Peaks were identified using external cannabinoid and terpene standards. Final values are given as milligrams of metabolite per gram of the original dried material.

### Statistical Analysis

Data were analyzed using unpaired Student’s *t*-tests. Analysis of variance (ANOVA) with a Tukey’s honest significant difference *post-hoc* test was used to assess differences in phytochemical content. A chi-square test was used to compare rooting success. All tests were conducted at *p* < 0.05 in the statistics program R (version 3.5.1). Graphs were plotted using Excel 2013.

## Results

### Survival Rate and Ploidy Determination

**FIGURE 1:**
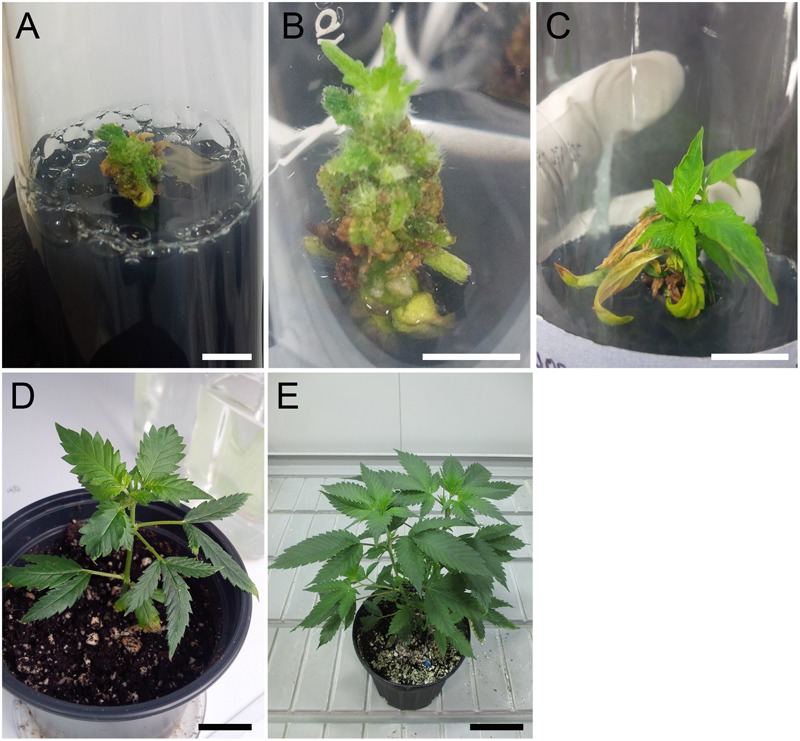
Regeneration of tetraploid shoots for *C. sativa* strain 2 following oryzalin treatment of axillary bud explants. **(A)** Deformed meristem structure at 5 weeks after oryzalin treatment. Scale bar, 5 mm. **(B)** Shoot initiation at 9 weeks. Scale bar, 5 mm. **(C)** Recovered shoot at 14 weeks. Scale bar, 15 mm. **(D)** Plantlet acclimatizing to soil at 19 weeks after treatment. Scale bar, 2 cm. **(E)** Mature tetraploid plant at 24 weeks. Scale bar, 8 cm.

Oryzalin is a potent herbicide that inhibits microtubule polymerization to promote polyploidization (Morejohn et al., 1987). Two *C. sativa* strains were tested: one THC dominant indica strain (strain 1), and one balanced THC/CBD indica-dominant hybrid strain (strain 2). Axillary buds treated with high concentrations of oryzalin had a poor survival rate. No explants survived the 150 μM treatment. Survival rates for explants treated with 20 μm oryzalin ranged from 62.5% to 87.5% for strain 1 and 2, respectively (Table 1). The majority of surviving shoots had small, curled leaves and deformed meristems. These structures persisted for several weeks before recovering and initiating small shoots (Figure 1). Flow cytometry analysis determined that nearly all the surviving shoots were successfully transformed. Of these, a large portion were mixoploid (73.3% and 46.7% for strains 1 and 2, respectively). Among the different treatments, 20 and 40 μM oryzalin had the best survival rates and produced the greatest number of tetraploids (Table 1). Overall, two tetraploid shoots were generated from strain 1 axillary buds and eight tetraploid shoots were generated from strain 2 axillary buds. While strain 2 tetraploid shoots recovered in culture and rooted normally, strain 1 tetraploid shoots grew poorly and failed to root. No further analysis was conducted on the strain 1 plants.

**Table 1:** Effect of oryzalin concentration on survival and polyploidization of *C. sativa* axillary bud explants treated for 24 h.

Oryzalin treatment (μM)	Strain 1 (High THC/Low CBD)				Strain 2 (Balanced THC/CBD)			
	No. of	Survival	Mixoploid	Tetraploid	No. of	Survival	Mixoploid	Tetraploid
	explants	rate (%)	plants (%)	plants (%)	explants	rate (%)	plants (%)	plants (%)
0	10	50	0	0	10	20	0	0
50	10	50	80	0	10	20	50	50
100	10	0	0	0	10	10	100	0
150	10	0	0	0	10	0	0	0
0	8	87.5	0	0	8	100	0	0
20	8	62.5	80	0	8	87.5	42.9	57.1
40	8	37.5	33.3	66.7	8	50	50	50
60	8	25	100	0	8	12.5	0	100

**FIGURE 2:**
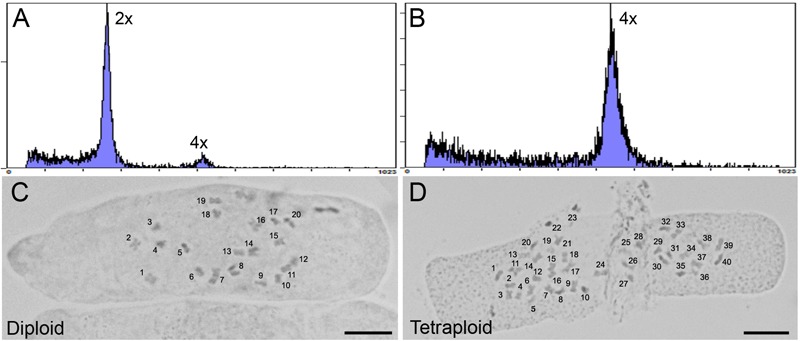
Analysis of ploidy by flow cytometry and root tip squash. **(A,B)** Flow cytometric histograms of the nuclear DNA content in diploid (2×) and **(B)** tetraploid (4×) leaf samples for *C. sativa* strain 2 plants, respectively. Y-axis, counts. X-axis, channel. **(C,D)** Root tip cells stained with 2% acetocarmine to observe chromosomes in diploid (2*n* = 2× = 20) and tetraploid (2*n* = 4× = 40) *C. sativa* strain 2 plants, respectively. Chromosomes are numbered for clarity. Scale bars, 10 μm.

One representative strain 2 tetraploid clone was selected for further analysis. Flow cytometry was used to determine a 2C nuclear DNA content of 3.93 ± 0.23 pg (*n* = 3) for the tetraploid, almost exactly twice the 1.97 ± 0.04 pg (*n* = 3) nuclear DNA content of the non-treated diploid mother plant (Figure 2A,B). The ploidy level of the plants was confirmed by determining the chromosome number in root tip squashes. These data showed that tetraploid cells contained 2*n* = 4× = 40 chromosomes compared to 2*n* = 2× = 20 chromosomes in diploid cells (Figure 2C,D). The ploidy of the tetraploid clone and its progeny were assessed several times showing that ploidy was stable following transfer to soil and propagation through cuttings for phenotype analysis.

### Tetraploid Phenotype

Significant effects of ploidy were noted on plant growth and morphology. To generate material for this analysis, diploid and tetraploid strain 2 plants in tissue culture were transferred to soil and grown into mother plants. Fifteen cuttings per mother plant were rooted in soil for phenotypic assessment and chemical analysis.

**FIGURE 3:**
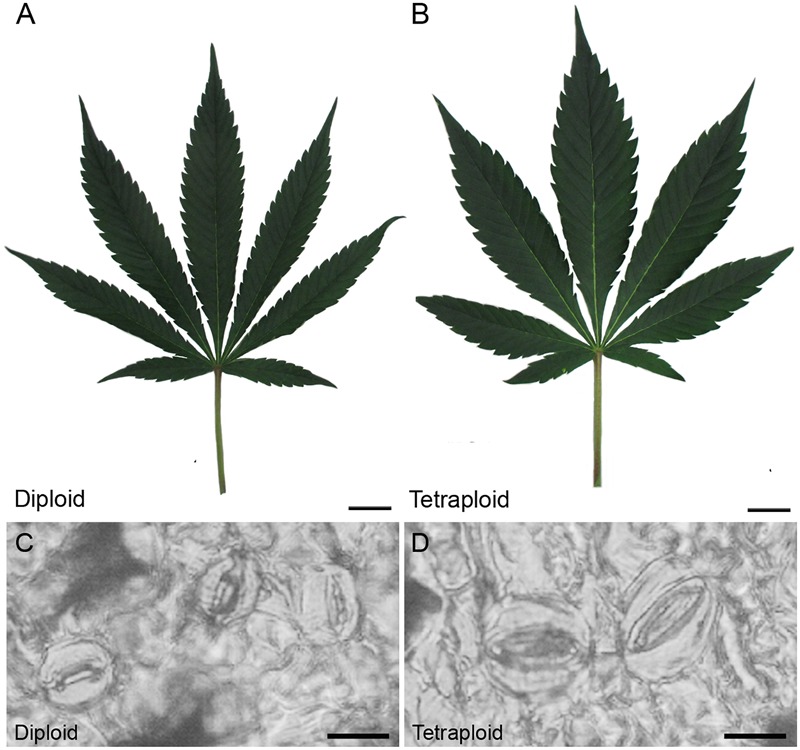
Leaf and stomata morphology. Representative images showing mature fan leaves of **(A)** diploid and **(B)** *C. sativa* strain 2 collected after 4 weeks of vegetative growth and 1 week under flowering lights. Scale bars, 2.5 cm. Nail polish impressions showing stomata on the abaxial surface of **(C)** diploid and **(D)** tetraploid fan leaves. Scale bars, 12 μm.

**FIGURE 4:**
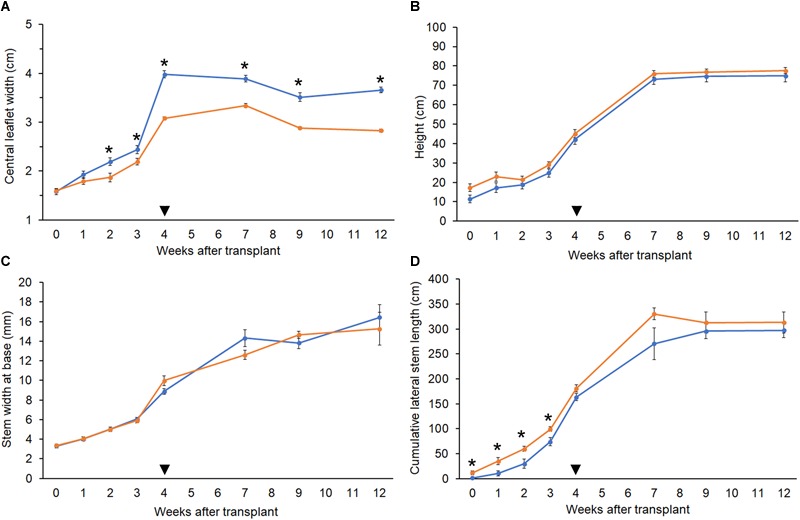
Growth parameters. Comparison of growth metrics in diploid (orange, *n* = 10) and tetraploid (blue, *n* = 9) *C. sativa* strain 2 plants. 5-week-old rooted clones were transplanted at week 0. Plants were moved to the flowering room at week 4 (arrowhead). Flowering lights were applied in week 5. **(A)** Width of the central leaflet in mature fan leaves. **(B)** Plant height from soil to highest meristem. **(C)** Diameter of the stem at 1 inch above the soil. **(D)** Sum of the length of all lateral stems. Data are means ± standard error. Asterisks indicate significant differences (Student’s *t*-test, *p* < 0.05).

The polyploid strain showed a reduction in rooting success. After 4 weeks, only 60% of tetraploid clones were successfully rooted (*n* = 9) compared to 100% of diploids (*n* = 15). Among rooted tetraploids, root emergence was slightly delayed (16.0 ± 3.7 days) compared to diploids (13.5 ± 4.7 days). Ploidy effects on leaf morphology were also observed. Tetraploids had larger fan leaves compared to diploids (Figure 3A,B). The central leaflet was significantly wider by an average of 0.75 cm on tetraploid leaves compared to diploid leaves, during the flowering phase (Figure 4A). Nail polish impressions showed that stomata on the underside tetraploid fan leaves were about 30% larger and half as dense compared to diploids (Table 2 and Figure 3C,D).

**Table 2:** Stomata size and density (mean ± SE) were measured on the abaxial side of mature fan leaves of diploid and tetraploid strain 2 *C. sativa* plants.

Ploidy	Stomatal Density	Guard Cell Length	Guard Cell Width
	(mm^2^)	(μm)	(μm)
Diploid	552.1 ± 18.2^a^ (*n* = 8)	16.0 ± 0.5^a^ (*n* = 24)	4.5 ± 0.1^a^ (*n* = 48)
Tetraploid	256.2 ± 18.9^b^ (*n* = 8)	21.7 ± 0.5^b^ (*n* = 24)	5.9 ± 0.1^b^ (*n* = 48)

*Means with different letters are significantly different (Student’s *t*-test, *p* < 0.05).*

The height and stem base width of diploid and tetraploid plants were similar throughout growth. During the vegetative phase, tetraploid plants had slightly shorter lateral stems, but this difference was not significant following the switch to flowering (Figure 4B–D). Plants of both ploidies showed their first flowers after 1 week under flowering lights, and the rate of floral growth was similar throughout the flowering phase.

**FIGURE 5:**
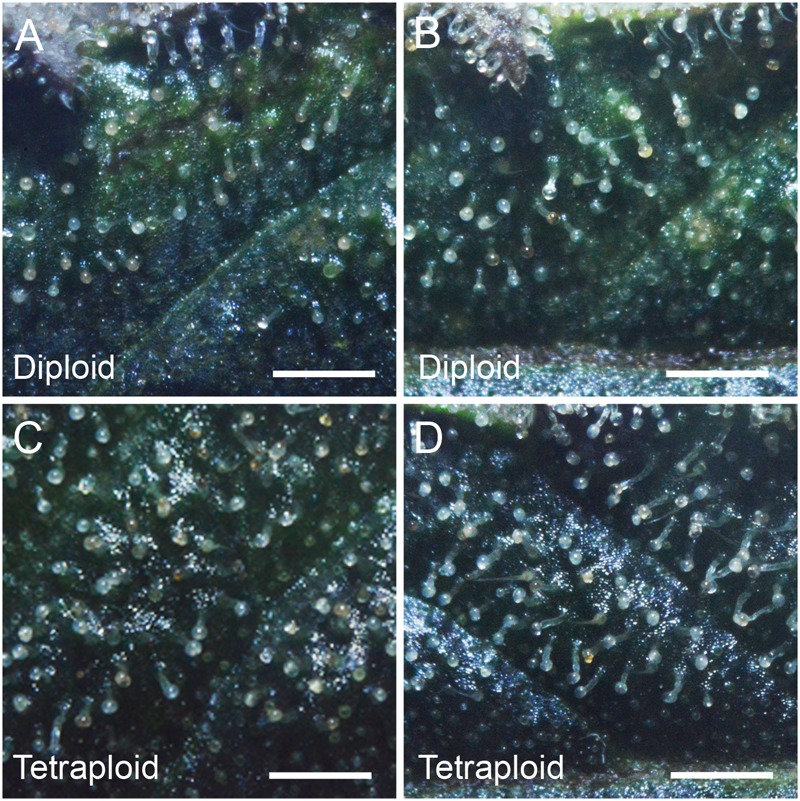
Trichome density. Representative images showing trichome density on the adaxial surface of the 4th sugar leaf of *C. sativa* strain 2 plants **(A,B)** diploid, **(C,D)** tetraploid. Leaves were imaged on the 7th week of flowering. Scale bars, 1 mm.

Trichome density on sugar leaves was measured at 2 weeks prior to harvest. Tetraploid leaves showed 40.4% higher glandular trichome density (4.41 ± 0.16 trichomes per mm^2^) compared to diploids (3.14 ± 0.15 trichomes per mm^2^). However, there was no obvious difference in the maturity of the trichomes on leaves, with the majority in the milky stage and some beginning to turn amber (Figure 5).

**FIGURE 6:**
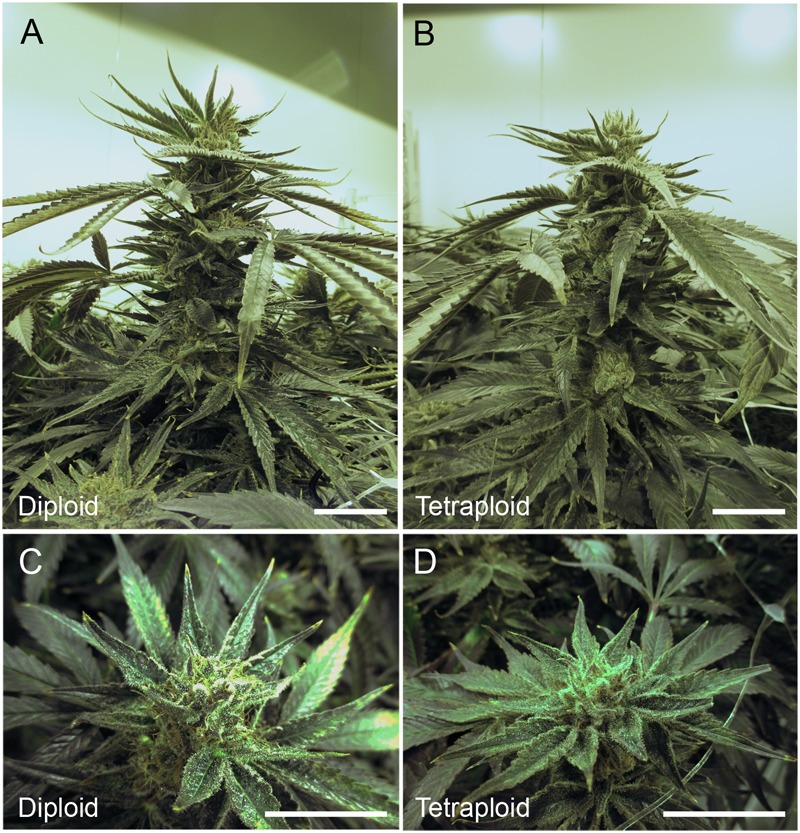
Inflorescence architecture. Representative images showing the cola (inflorescence apex) and buds of *C. sativa* strain 2 plants during the 8th week of flowering (week 12 after transplanting and 1 week before harvesting). Cola for **(A)** diploid and **(B)** tetraploid. Scale bars, 5 cm. Close-ups showing bud morphology for **(C)** diploid. Scale bar, 1.5 cm. **(D)** tetraploid. Scale bar, 2.5 cm.

The inflorescence apex and bud morphologies were similar for plants of both ploidies (Figure 6). Tetraploid yields trended higher at harvest, but there was no significant difference in whole plant weight, weight of trimmed bud (buds trimmed of excess leaves) or trim weight (leaf trimmings) of diploids versus tetraploids (Table 3). Further, there was no significant difference in the final dry weight of buds, which averaged 38.0 ± 6.4 g per plant for tetraploids and 34.3 ± 5.8 g per plant for diploids. These data indicate that chromosome doubling had no significant effect on plant growth, maturity, or yield.

**Table 3:** Yield metrics (mean ± SE) of strain 2 *C. sativa* plants after 4 weeks of vegetative growth and 8 weeks of flowering (*n* = 10 for diploids, *n* = 9 for tetraploids).

Ploidy	Weight (g)			
	Whole plant	Wet bud	Leaf trim	Dry bud
Diploid	527.78 ± 76.66^a^	134.50 ± 16.40^a^	145.60 ± 19.63^a^	34.35 ± 5.76^a^
Tetraploid	529.78 ± 99.22^a^	180.44 ± 30.90^a^	201.89 ± 37.95^a^	38.00 ± 6.37^a^

*All tissue measured wet, except dry bud. Means with different letters are significantly different (Student’s *t*-test, *p* < 0.05).*

### Phytochemical Content

**FIGURE 7:**
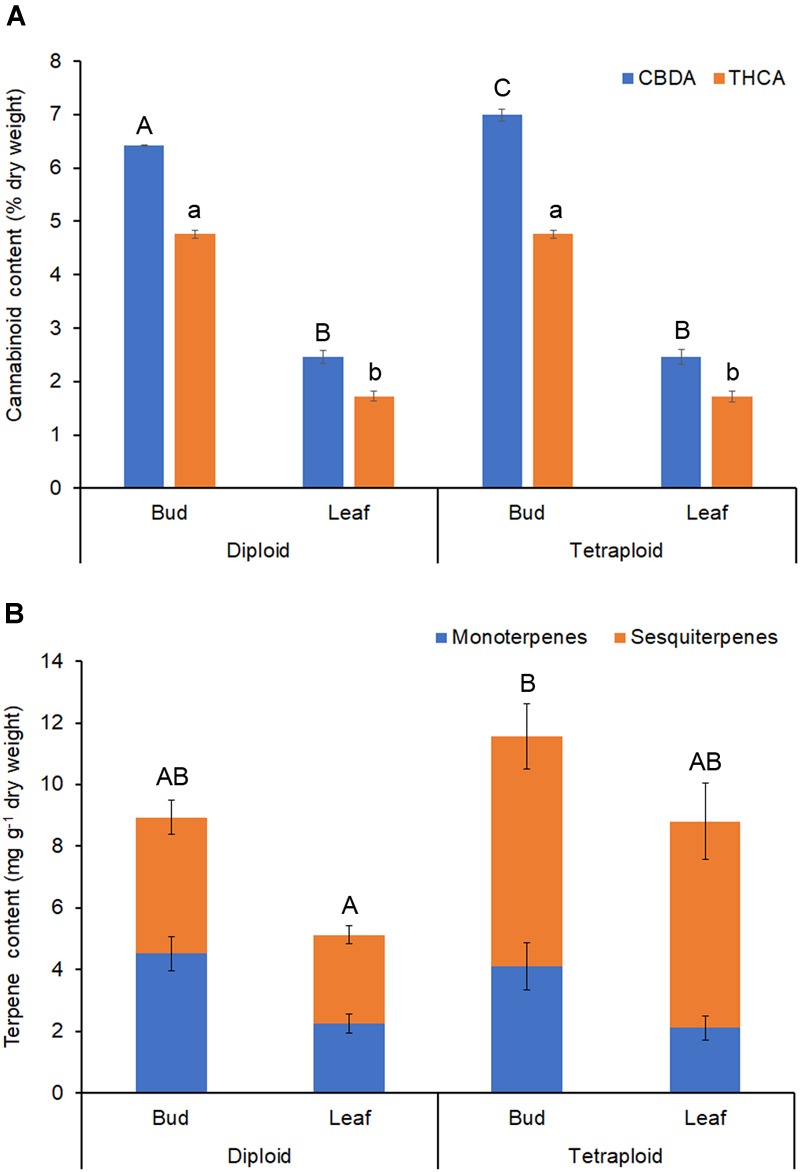
Phytochemical content. Dried buds and leaves of strain 2 *C. sativa* plants of different ploidy were assessed by HPLC and GC for cannabinoids and terpenes, respectively. **(A)** Cannabinoid profile. **(B)** Terpene profile. Data are means ± standard error (*n* = 10 for diploids, *n* = 9 for tetraploids, cannabinoid samples analyzed in duplicate). Means (CBDA/THCA/total terpenes) with different upper/lower-case letters are significantly different (ANOVA with Tukey’s *post hoc* test, *p* < 0.05).

Δ^9^-tetrahydrocannabinol and CBD are the main active ingredients in Cannabis, which in plants are mainly found in their acid forms (Andre et al., 2016). HPLC analysis showed that the ratio of THCA to CBDA was similar in strain 2 diploids and tetraploids, with about 35% more CBDA than THCA (Table 4 and Figure 7A). Overall, the major cannabinoids comprised 64.16 ± 0.98 mg g^-1^ CBDA and 47.56 ± 0.70 mg g^-1^ THCA in the diploid buds, and 69.89 ± 1.12 mg g^-1^ CBDA and 47.56 ± 0.76 mg g^-1^ THCA in the tetraploid buds (Table 4). These values represent a significant 8.9% increase in CBDA in buds. No corresponding increase in THCA was found. Significant changes were also noted in the buds for some of the minor cannabinoids: a 34.3% reduction in cannabigerolic acid and a 15.2% increase in cannabidivarinic acid. No cannabinol, cannabicyclol, or Δ^8^-tetrahydrocannabinol (breakdown products) were detected in leaves or buds, and cannabidivarin was absent from the leaves. As expected, leaves had a significantly lower cannabinoid content, totaling about 35% the concentration of the buds (Table 4 and Figure 7A).

**Table 4:** Cannabinoid content (mean ± SE) for dried leaf and bud material of diploid and tetraploid strain 2 *C. sativa* plants analyzed in duplicate (*n* = 10 for diploids, *n* = 9 for tetraploids) by HPLC.

Metabolite	Content (mg/g dried tissue)			
	Diploid bud	Diploid leaf	Tetraploid bud	Tetraploid leaf
Cannabidiol	2.50 ± 0.10^a^	1.03 ± 0.04^b^	2.94 ± 0.15^c^	1.28 ± 0.07^b^
Cannabidiolic acid	64.16 ± 0.98^a^	22.46 ± 1.20^b^	69.89 ± 1.12^c^	24.58 ± 1.38^b^
Δ^9^-tetrahydrocannabinol	2.82 ± 0.09^a^	1.26 ± 0.05^b^	3.41 ± 0.12^c^	1.55 ± 0.08^b^
Δ^9^-tetrahydrocannabinolic acid	47.56 ± 0.70^a^	17.20 ± 0.92^b^	47.56 ± 0.76^a^	17.23 ± 1.01^b^
Cannabinol	0^a^	0^a^	0^a^	0^a^
Cannabigerol	0.48 ± 0.01^a^	0.06 ± 0.02^b^	0.41 ± 0.01^c^	0.01 ± 0.01^b^
Cannabigerolic acid	1.46 ± 0.08^a^	0.33 ± 0.02^b^	0.96 ± 0.01^c^	0.28 ± 0.04^b^
Δ^8^-tetrahydrocannabinol	0^a^	0^a^	0^a^	0^a^
Cannabichromene	0.24 ± 0.07^a^	0^b^	0.12 ± 0.01^ab^	0.05 ± 0.03^bc^
Cannabicyclol	0^a^	0^a^	0^a^	0^a^
Cannabidivarin	0.01 ± 0.01^a^	0^a^	0.02 ± 0.01^a^	0^a^
Cannabidivarinic acid	0.33 ± 0.01^a^	0^b^	0.38 ± 0.01^c^	0^b^
Total cannabinoids	119.6 ± 1.81^a^	42.30 ± 2.22^b^	125.70 ± 2.10^a^	45.00 ± 2.50^b^

*Means with different letters are significantly different for measurements of a single cannabinoid (ANOVA with Tukey’s *post hoc* test, *p* < 0.05).*

Terpenes that contribute to the taste and aroma of Cannabis products are mainly monoterpenes and sesquiterpenes (Andre et al., 2016). Tetraploids showed an increase in the overall terpene content of leaves (Table 5 and Figure 7B). Total leaf terpenes were increased by 71.5% bringing the total terpene content to 8.8 ± 1.26 mg g^-1^ which was similar to the diploid buds. Tetraploid buds also had increased total terpene content, which reached 11.58 ± 1.78 mg g^-1^. However, due to high individual variation between plants, these differences were not statistically significant (Table 5). Specific terpenes showed significant changes. In buds and leaves, the monoterpene limonene was significantly lower, whereas the sequiterpene *cis*-nerolidol was significantly increased, comprising up to 3.50 mg g^-1^ in tetraploid buds. Overall, greater accumulation of sesquiterpenes was responsible for the increased terpene content of tetraploid leaves and buds (Table 5 and Figure 7B). Tetraploid buds showed a 60% increase in guaiol. Tetraploid leaves also showed double the amount of sesquiterpene α-humulene and contained α-bisabolol, which was absent in the diploid leaves (Table 5).

**Table 5:** Terpene content (mean ± SE) in the dried leaf and bud material of diploid and tetraploid Strain 2 *C. sativa* plants (*n* = 10 for diploids, *n* = 9 for tetraploids) by gas chromatography.

Metabolite	Terpene class	Content (mg/g dried tissue)			
		Diploid bud	Diploid leaf	Tetraploid bud	Tetraploid leaf
α-Pinene	monoterpene	1.06 ± 0.13^a^	0.51 ± 0.07^b^	1.03 ± 0.14^a^	0.56 ± 0.11^b^
Camphene	monoterpene	0^a^	0^a^	0^a^	0^a^
β-Pinene	monoterpene	0.51 ± 0.07^a^	0.21 ± 0.03^b^	0.41 ± 0.06^a^	0.20 ± 0.05^b^
Myrcene	monoterpene	2.29 ± 0.25^a^	1.11 ± 0.13^bc^	1.74 ± 0.23^ab^	0.87 ± 0.16^c^
Δ-3-Carene	monoterpene	0^a^	0^a^	0^a^	0^a^
α-Terpinene	monoterpene	0^a^	0^a^	0^a^	0^a^
p-Cymene	monoterpene	0^a^	0^a^	0.01 ± 0.01^a^	0^a^
Limonene	monoterpene	0.24 ± 0.06^a^	0.13 ± 0.04^ab^	0.06 ± 0.04^b^	0.01 ± 0.01^b^
Eucalyptol	monoterpene	0^a^	0^a^	0^a^	0^a^
Ocimene	monoterpene	0^a^	0^a^	0.05 ± 0.05^a^	0^a^
γ-Terpinene	monoterpene	0^a^	0^a^	0^a^	0^a^
Terpinolene	monoterpene	0^a^	0^a^	0.01 ± 0.01^a^	0^a^
Linalool	monoterpene	0.34 ± 0.03^a^	0.23 ± 0.03^a^	0.38 ± 0.09^a^	0.25 ± 0.04^a^
Isopulegol	monoterpene	0^a^	0^a^	0.12 ± 0.11^a^	0.03 ± 0.03^a^
Geraniol	monoterpene	0^a^	0^a^	0.27 ± 0.18^a^	0.15 ± 0.09^a^
α-Terpineol	monoterpene	0.08 ± 0.03^a^	0.06 ± 0.03^a^	0.03 ± 0.01^a^	0.01 ± 0.00^a^
g-Terpineol	monoterpene	0^a^	0^a^	0^a^	0^a^
β-Caryophyllene	sesquiterpene	1.35 ± 0.06^a^	1.07 ± 0.06^a^	1.56 ± 0.19^a^	1.52 ± 0.24^a^
α-Humulene	sesquiterpene	0.48 ± 0.03^ab^	0.35 ± 0.04^b^	0.86 ± 0.20^a^	0.72 ± 0.12^ab^
cis-Nerolidol	sesquiterpene	2.12 ± 0.31^ab^	1.44 ± 0.24^a^	3.50 ± 0.41^b^	3.16 ± 0.51^b^
trans-Nerolidol	sesquiterpene	0^a^	0^a^	0.51 ± 0.38^a^	0.18 ± 0.18^a^
Guaiol	sesquiterpene	0.05 ± 0.01^ab^	0.04 ± 0.01^a^	0.08 ± 0.01^b^	0.07 ± 0.02^ab^
α-Bisabolol	sesquiterpene	0.41 ± 0.21^ab^	0^a^	0.97 ± 0.25^b^	1.04 ± 0.41^b^
Total monoterpenes		4.52 ± 0.55^a^	2.24 ± 0.31^b^	4.10 ± 0.76^ab^	2.11 ± 0.38^b^
Total sesquiterpenes		4.42 ± 0.55^ab^	2.89 ± 0.30^a^	7.47 ± 1.05^b^	6.70 ± 1.25^b^
Total Terpenes		8.94 ± 0.36^ab^	5.13 ± 0.39^a^	11.58 ± 1.78^b^	8.80 ± 1.26^ab^

*Means with different letters are significantly different for measurements of a single terpene (ANOVA with Tukey’s *post hoc* test, *p* < 0.05).*

## Discussion

Ploidy manipulation is a valuable tool in plant breeding. Important consequences of genome doubling can include larger organs and improved production of secondary metabolites, often linked to increased tolerance to biotic and abiotic stress. Polyploid forms also provide a wider germplasm base for breeding (Meru, 2012; Sattler et al., 2016). Polyploids have yet to be implemented in most breeding programs for Cannabis.

Here, we show that treatment of axillary buds with the dinitroaniline herbicide oryzalin is an effective method for chromosome doubling. Past studies on the polyploidization of hemp (Bagheri and Mansouri, 2015; Mansouri and Bagheri, 2017) and its closest relative hops (*Humulus lupulus* L.) used colchicine for doubling (Roy et al., 2001; Trojak-Goluch and Skomra, 2013). However, oryzalin has greater specificity for plant tubulins (Morejohn et al., 1987) and is considered a more effective and less toxic alternative to colchicine (Petersen et al., 2003; Stanys et al., 2006; Ascough et al., 2008; Dhooghe et al., 2009; Sakhanokho et al., 2009; Viehmannová et al., 2009; Rêgo et al., 2011). Trojak-Goluch and Skomra (2013) found that 1250 μM of colchicine applied to explants was the most effective for polyploidization of hops. Shown here, concentrations in the range of 20 and 40 μM were the most effective for tetraploidization of Cannabis, indicating that oryzalin is effective at over 30 times lower concentration compared to colchicine. Strain 1 was less tolerant of oryzalin treatment compared to strain 2 and yielded a higher ratio of mixoploids. Similar genotype differences in response to oryzalin treatment have been found in other species such as cherry laurel and Japanese quince (Stanys et al., 2006; Contreras and Meneghelli, 2016). The two tetraploids of strain 1 that were isolated did not easily regenerate shoots on the current media. Compared to strain 2 tetraploids, these plants were sickly and slow-growing. This response could reflect a greater sensitivity to oryzalin treatment or polyploidization may alter media requirements or hormone concentrations necessary to grow shoots.

One representative strain 2 tetraploid was analyzed in this study. The ploidy of this strain proved stable through propagation in tissue culture and transfer to soil. Ploidy has also been stable throughout one generation of cloning. Seven subsequent strain 2 tetraploids were isolated (Table 1). All of these plants have shown stable ploidy to date. An eighth potential tetraploid was isolated but reverted to mixoploid status upon second analysis. It is possible that this plant was initially mixoploid with a small portion of diploid cells that quickly multiplied (Blakeslee and Avery, 1937; Stanys et al., 2006). Further testing will determine if the stability of tetraploid clones lasts over multiple generations and is preserved if plants are propagated through seeds.

Overall, clone health and survival was lower among tetraploid clones, possibly due to lower rooting success. This finding matches with hops, whose tetraploids also have slower root development in culture and difficulty acclimating to a greenhouse environment (Roy et al., 2001; Trojak-Goluch and Skomra, 2013). Despite these early difficulties, tetraploid strain 2 *C. sativa* plants grew and flowered at a rate comparable to diploids, yielding a similar amount of dried bud. Should this clone be representative, our data suggest that tetraploidization of Cannabis hinders rooting but has no significant negative effect on overall plant growth or yield.

A widespread consequence of polyploidy is an increase in cell size, caused by a larger number of gene copies. However, an increase in cell size does not always translate to increased size of the whole plant or its organs, since the number of cell divisions in polyploids can be reduced (Sattler et al., 2016). Measurements showed that the fan leaves of tetraploid Cannabis plants were significantly larger than diploids, most evident during the flowering phase. On the other hand, yield of dried bud was not higher, indicating no increase in floral size. Trojak-Goluch and Skomra (2013) found significant differences in cone weight between individual hops tetraploids, some of which were not significantly different from the diploid control. Analysis of additional tetraploid individuals may clarify whether or not polyploidization leads to increased floral size in Cannabis.

Stomata were also about 30% larger (length and width) and less than half as dense (46%) compared to diploid leaves. Tetraploids of hemp also exhibit a lower density of stomata and stomata guard cells with larger length and diameter, and leaves are shorter and wider compared to diploids (Mansouri and Bagheri, 2017). Changes in stomata size and density are common among tetraploids (Ascough et al., 2008; Sakhanokho et al., 2009; Rêgo et al., 2011; Talebi et al., 2017). Overall, these data suggest that stomata size and density are reliable phenotypic markers for polyploid Cannabis.

Phytochemical content is one of the most important factors to consider in Cannabis production. The major cannabinoids THC and CBD in acid form are produced from a common cannabigerolic acid precursor by THCA synthase and CBDA synthase, respectively (Andre et al., 2016). The cannabinoid ratio is determined by co-dominant alleles of these synthase enzymes, which occur at a single locus on chromosome 6 (De Meijer et al., 2003; Marks et al., 2009). A number of allellic variants of these enzymes exist in different cultivars, and each has a unique effect on cannabinoid production. Therefore, large-scale genome rearrangements or duplications such as polyploidization could enable new allelic combinations, which have the potential to create novel chemotypes (Laverty et al., 2018).

Chemical analysis of strain 2 tetraploids found little change in the cannabinoid profile relative to diploids. THCA content was similar and there was small but significant 8.9% increase of CBDA in tetraploid buds. The cannabigerolic acid precursor of cannabinoids is normally present at very low levels in the plant because of continual conversion to end products. Notably, tetraploids showed a significant ∼30% reduction in cannabigerol acid precursor. Linkage analysis suggests that availability of this precursor is a strong limiting factor in determining the overall yield of THC in plants (Laverty et al., 2018). Chemical analysis of tetraploid hemp found a 33% decrease in THC and little or no change in CBD content (Bagheri and Mansouri, 2015). These collective data suggest that ploidy may have limited influence on the cannabinoid biosynthetic pathway.

Terpenes are important aromatic compounds that determine the smell and taste of Cannabis products, and also modulate the drug effects of cannabinoids. Terpene concentrations above 0.5 mg g^-1^ are considered pharmacologically relevant (Russo, 2011). In the buds and leaves, two additional sesquiterpenes reached this threshold in tetraploids, both of which have been found to be potent anti-inflammatories: α-humulene and α-bisabolol (Fernandes et al., 2007; Passos et al., 2007; Maurya et al., 2014). α-bisabolol is also known to be analgesic, antibiotic, and can moderately enhance skin penetration of other compounds (Kamatou and Viljoen, 2010). Additionally, although *cis*-nerolidol was above the biological relevance threshold in both diploids and tetraploids, this terpene was increased an average of 1.92-fold in the tetraploids. Nerolidol is a sedative and can interact with THC to enhance relaxation effects (Russo, 2011). This compound also functions as an excellent skin penetrant, which would be beneficial for topical Cannabis preparations (Kamatou and Viljoen, 2010). Although there was a significant decrease in limonene, this monoterpene is not present at concentrations likely to be biologically active. However, changes in smell or taste, which were not assessed in this study, may result.

Overall, total terpene content was increased in the leaves and buds of tetraploid strain 2 plants. However, the increase did not reach statistical significance in either case. In general, terpene content was more variable in the tetraploids compared to diploids. This variability may be reflective of epigenetic instability which can occur in newly generated polyploids, resulting in greater variance between plants (Adams and Wendel, 2005; Comai, 2005). Sequiterpenes were primarily responsible for the terpene increase in leaves and buds, suggesting a significant effect of ploidy on the cytosolic malvalonic acid biosynthetic pathway for sequiterpenes. Monoterpenes, showing little change, come from a plastid-localized methyl-erythritol phosphate pathway whose geranyl diphosphate precursor is also a building block for cannabinoids (Flores-Sanchez and Verpoorte, 2008; Andre et al., 2016). A 71.5% increase in terpene content of leaves correlates well with increased trichome density on tetraploid sugar leaves. The terpene content of buds was also higher by about 30% suggesting that trichome density on flowers is also increased. It is unclear why the increase in trichomes did not also correlate with an increase in cannabinoids. A combination of factors may be important. Such is the case for *Artemisia annua*, where yield of the antimalarial compound artemisinin depends on leaf dry weight, availability of metabolic precursors, and efficiency of conversion to end products, in addition to trichome density (Lommen et al., 2008).

Although the phytochemical content of tetraploid material is lower in leaves than in buds, particularly for the cannabinoids, this content is high enough for the trimmed leaf material to be used for extraction. Notably, the terpenes were increased in the tetraploid leaves to the point where the total terpene content was comparable to the diploid bud. Considering that the wet trim weight was usually similar to, or slightly higher than, the bud yield, extraction of quality trim material could almost double total production yield. Even if cannabinoids are low in the tetraploid leaves, a terpene-rich extract would have many commercial applications, such as flavoring for Cannabis edibles or as independent products with novel therapeutic properties.

Results from this investigation, should they prove representative, indicate that tetraploid Cannabis plants grow normally – apart from reduced rooting – and have a similar chemical profile to diploids, with notable increases in CBD and sesquiterpenes. Despite these modest changes, synergistic interactions between the various components may in fact result in an altered biological response to this product, particularly since CBD and the terpenes can modify the activity of THC (Russo, 2011). The key development in this study was the establishment of an efficient method of producing polyploids in Cannabis, laying the groundwork for larger scale production and assessment of tetraploids and downstream breeding of improved Cannabis varieties for both the medical and recreational industries.

## Author Contributions

SH and EB developed the initial experiment proposal and helped with method development. GG optimized tissue culture methods for Cannabis. TJ and SM conducted flow cytometry analysis and assisted with root tip squash method optimization. JP developed oryzalin treatment and other methods and carried out the laboratory work, and phenotype analysis. JP, SH, EB, and SM assisted with writing and editing.

## Conflict of Interest Statement

JP, GG, and EB are employees of Canopy Growth Corporation. There is a patent pending for the method of polyploidization in Cannabis. The remaining authors declare that the research was conducted in the absence of any commercial or financial relationships that could be construed as a potential conflict of interest.
